# Influence of Vaccination Characteristics on COVID-19 Vaccine Acceptance Among Working-Age People in Hong Kong, China: A Discrete Choice Experiment

**DOI:** 10.3389/fpubh.2021.793533

**Published:** 2021-12-10

**Authors:** Kailu Wang, Eliza Lai-Yi Wong, Annie Wai-Ling Cheung, Peter Sen-Yung Yau, Vincent Chi-Ho Chung, Charlene Hoi-Lam Wong, Dong Dong, Samuel Yeung-Shan Wong, Eng-Kiong Yeoh

**Affiliations:** ^1^Centre for Health Systems and Policy Research, JC School of Public Health and Primary Care, Faculty of Medicine, The Chinese University of Hong Kong, Hong Kong, China; ^2^JC School of Public Health and Primary Care, Faculty of Medicine, The Chinese University of Hong Kong, Hong Kong, China

**Keywords:** COVID-19 vaccine, vaccine hesitancy, quarantine measures, conjoint analysis, discrete choice experiment, peer influence

## Abstract

**Background:** Along with individual-level factors, vaccination-related characteristics are important in understanding COVID-19 vaccine hesitancy. This study aimed to determine the influence of these characteristics on vaccine acceptance to formulate promotion strategies after considering differences among respondents with different characteristics.

**Methods:** An online discrete choice experiment was conducted among people aged 18–64 years in Hong Kong, China, from 26 to 28 February 2021. Respondents were asked to make choices regarding hypothetical vaccination programmes described by vaccination-related characteristics—the attributes derived from a prior individual interview. Subgroup analysis was performed to identify the differences in vaccination-related characteristics among respondents with different personal characteristics.

**Results:** A total of 1,773 respondents provided valid responses. The vaccine efficacy and brand were the most important factors affecting acceptance, followed by the exemption of quarantine for vaccinated travelers, safety, venue for vaccination, vaccine uptake of people in their lives, and recommendations by general physicians or government. Frequent exposure to vaccination information on social media has been associated with increasing vaccine refusal. Substantial preference heterogeneity for the attributes was found among people of different ages, incomes, chronic conditions, and previous acceptance of influenza vaccines.

**Conclusion:** The findings provided evidence to formulate interventions to promote vaccine uptake, including the provision of vaccination at housing estate or workplaces, involvement of general physicians and interpersonal communication in vaccine promotion and information dissemination, and exemption of quarantine for vaccinated people. Moreover, social media is a significant information channel that cannot be neglected in the dissemination of official information.

## Introduction

Although multiple non-pharmaceutical interventions and measures, including social distancing, use of facemasks, and border control have reduced the risk of COVID-19 spread (1, 2), effective and efficient vaccination remains a crucial method to prevent the transmission of SARS-CoV-2 (3). By the end of March 2021, ~12 vaccines were available for emergency use (4). In administering vaccination, its coverage and the rollout speed are as important as the efficacy to achieve greater effectiveness in disease prevention (5, 6), for it was estimated that the coverage needed to achieve 60–72% to generate herd immunity for the population using a vaccine with 100% efficacy (7).

However, vaccine hesitancy poses a great threat to achieving this goal, as it could lead to delay or refusal among vaccinated people who perceived vaccination as unsafe or unnecessary, and this has increased globally over the years (8, 9). This problem is crucial for Hong Kong. In February 2021, the Hong Kong government confirmed that the vaccination is free of charge and provided choices for the vaccines. Three vaccines were planned to be launched. There were not many difficulties in scheduling vaccination, due to sufficient supply and the launch of multiple vaccination centers all over the city. Since the study was conducted at beginning of the vaccination programme for local population, no incentives had been formulated to increase the rate during the survey period. Previous studies conducted among the aforementioned population found a low acceptance rate for COVID-19 vaccines (34.8–37.2%) compared to other countries and regions where the rate ranged from ~55 to 90% (10–12). On 9 April 2021 (1.5 months after commencement of vaccination), the vaccination coverage was around 9.3 doses per 100 people, and the daily number of people receiving the first dose was reduced to around half of the people receiving the second dose since the end of March 2021 (13).

Numerous studies have been conducted to identify the individual-level factors associated with vaccine hesitancy among different subpopulations across the world, including age, ethnicity, education level, perceived risk of infection, and previous acceptance of other vaccines (11, 14–20). Fewer studies have estimated the influence of vaccine characteristics and vaccination delivery. Several discrete choice experiments or conjoint analyses which allow a more realistic and natural setting than the consideration of single factors, have been conducted in Australia, mainland China, France, the US, and the UK (21–29). Consistent findings show the efficacy of the vaccine in disease prevention and the place of origin as the most important factors, while the other factors, including safety, duration of immunity, number of doses needed, and venue for vaccination had less influence (21–29). While these studies have reported the influence of a few vaccine characteristics, including efficacy, safety, immunity duration, number of doses, and place of origin, there are a few modifiable factors that can be useful in further studies for discussion, such as the arrangement of vaccination programmes, the benefits after vaccination, and the recommendations of healthcare workers. For example, a study in the US reported that the provision of vaccination proof could improve willingness (29), and a UK study found that the recommender and venue of the vaccination affect vaccination intentions (23). Therefore, the effect of additional modifiable factors should be explored and examined to inform the design of interventions and policies to promote COVID-19 vaccination.

In light of these, there is a need to determine the relative importance among the vaccination-related characteristics, especially those that can be modified or translated into interventions in promoting COVID-19 vaccine acceptance and uptake, and find whether this impact is different across people with different sociodemographic and health-related characteristics. We hypothesized that (1) willingness to accept the COVID-19 vaccine is associated with individual-level characteristics of the respondents, including education, occupation, income, chronic condition, perceived risks and severity, previous influenza vaccination, and information sources, based on the findings from previous studies on associated between individual-level factors and vaccine acceptance or hesitancy (11, 14–20); (2) the attribute levels for the COVID-19 vaccine have significant effects on acceptance; and (3) the attribute levels for vaccination plans also have significant effects on acceptance. The findings of this study could provide evidence and insights for government, policymakers, and healthcare professionals to formulate and tailor promotion strategies for COVID-19 vaccination.

## Methods

A cross-sectional questionnaire survey incorporated with a discrete choice experiment (DCE) was conducted among a Chinese population aged 18–64 years in Hong Kong from 26 to 28 February 2021 1 week after the vaccination programme for COVID-19 was launched for local priority groups.

### Study Sample and Data Collection

This study targeted the working-age Chinese population living in Hong Kong, the key population to reach herd immunity and revive the economy. Adults aged 18–64 years who were Hong Kong residents (i.e., only residents who were eligible for COVID-19 vaccination in Hong Kong) were included in the study. Those who were diagnosed with COVID-19 or who had already received the COVID-19 vaccine were excluded from the study sample. The respondents were recruited using a convenience sampling approach and consisted of a well-stratified sample according to the working population profile in Hong Kong (30). Minimum sample size was estimated based on the formula provided by a previous DCE study as below (22).


(1) $$\begin{array}{r} \text{n}=\frac{1-p}{p\cdot k\cdot d^{2}}\times {\left[\Phi^{-1}\left(1-\frac{\propto }{2}\right)\right]}^{2} \end{array}$$

Where *p* refers to the population probability of interests (in this case, COVID-19 vaccination acceptance rate), k is the number of choice tasks for each respondents, d is the precision of the estimation of the population probability, α is the significance level, and Φ^−1^ is the inverse cumulative function of normal distribution. Based on previous local studies (10, 11), acceptance rate of COVID-19 vaccine was 34.8–37.2%. To be conservative, *p* = 30% was used in the estimation. There are k = 8 tasks for each respondent, and we aimed to derive an estimate for *p* within 3% (d) of the true probability with 95% confidence level. The minimum sample size should be 1,245. Assuming 50% would response to the questionnaire, we targeted to recruit around 2,490 persons with at least 1,245 valid responses for the DCE.

The questionnaire was completed in Chinese. A link to the online self-administered questionnaire was sent to the potential respondents using the contact information of participants from a panel of respondents in different occupation groups of previous surveys (31), as well as through non-governmental organizations that provide social services to people in lower socioeconomic groups such as income and occupation, to ensure heterogeneity of working population as they were more difficult to approach based on previous experience. The distributions of age, occupation groups, and socioeconomic class among the study sample were assessed during the implementation of the survey to adjust the strategies for sending the survey to the remaining participants. In the questionnaire, an informed sheet and an electronic consent form were available for respondents. This was followed by several questions screening for eligibility based on the inclusion criteria. The data collected by the questionnaire were password-protected and retrieved from the University online server for further processing.

### Attributes of DCE

The DCE, also known as choice-based conjoint analysis, involves choices made by respondents between alternatives in each of the choice sets, which are described by vaccination-related characteristics (i.e., vaccination attributes) (32). The vaccination attributes for this study included vaccine brand (Sinovac [inactivated vaccine], BioNTech/Fosun [mRNA vaccine], AstraZeneca [viral vector vaccine]); efficacy [50, 70, 90%, based on the efficacy reported by relevant clinical trials (33–36)]; probability of serious adverse events [1 out of 100,000 people, 1 out of 10,000 people, defined as the events that could cause death, life-threatening conditions, disability and/or hospitalization. The levels are determined based on the rate of serious adverse event reported by relevant clinical trials and DCE study (22, 33–36)]; and modifiable attributes, including vaccine uptake of people in their lives (family members, friends/colleagues), recommendations from professionals (government expert advisory panels, and general physicians); venue for vaccination (community halls, housing estate/workplace, and healthcare facilities); and quarantine arrangements for vaccinated travelers (compulsory 14-day quarantine, exemption of 14-day quarantine) (Table 1). These attributes were derived from individual interviews of 45 adults living in Hong Kong with diverse socio-demographic characteristics regarding age, sex, education, and chronic conditions, guided by the Theoretical Domains Framework (TDF). TDF is an integrative framework with 14 domains synthesizing several theories in behavioral change and is commonly used for cross-disciplinary behavioral research (37). The interview findings indicated that the key factors that affect people's willingness to receive the vaccine included expected resumption to normal life, the origins or brands of the vaccine, perceived benefits and importance of receiving the vaccination, concerns of side effects, recommendations from healthcare professionals, the travel distance to vaccination locations, and influences of others' suggestions. The vaccine brand was included as an attribute in addition to efficacy and safety as it has been frequently mentioned in the prior qualitative study, and we aimed to examine the preference for brand independently from its efficacy and safety characteristics. Details of generating the attributes and levels can be found in Supplementary Material 1.

**Table 1:** The attributes and levels of the DCE.

**Attributes**	**Level 1**	**Level 2**	**Level 3**
Brand	Sinovac	BioNTech/Fosun	AstraZeneca
Probability of COVID-19 infection (efficacy)	Reduce 50%	Reduce 70%	Reduce 90%
Probability of serious adverse event (safety)	1 out of 100,000	1 out of 10,000	–
Vaccine uptake of people around	No known people received the vaccine	Family members received the vaccine	Friends/colleagues received the vaccine
Recommendations from professionals	Recommended by the expert advisory panel of the government	Recommended by the general physicians	–
Venue for vaccination	Community center	Housing estate/workplace	Healthcare facilities
Quarantine for vaccinated travelers	At least 14-day compulsory quarantine	The 14-day compulsory quarantine can be exempted	–

### DCE and Questionnaire Design

This DCE was designed using the D-optimality algorithm with zero prior means of the main effects in Stata 15.0 to select 32 pairwise choice sets from combinations of attribute levels generated from the full factorial design. To reduce the cognitive burden on the respondents, the 32 choice sets were assigned to four blocks, each consisting of eight choice sets; therefore, each respondent was randomly assigned to a block and had to answer only eight questions. The selected choice sets were screened to determine whether they presented realistic vaccination alternatives, and no manual alteration was required. An opt-out option for “accept neither vaccination programme” was added to each choice set to record vaccine refusal behaviors. An example of the choice sets is shown in Table 2.

**Table 2:** Example of the choice sets in the survey.

	**Vaccination plan 1**	**Vaccination plan 2**	**Do not receive both plans**
Brand	BioNTech/Fosun	AstraZeneca	None
Probability of COVID-19 infection (efficacy)	Reduce 90%	Reduce 70%	No reduction
Probability of serious adverse event (safety)	1 out of 10,000	1 out of 100,000	No serious adverse event
Vaccine uptake of people around	Family members received the vaccine	No known people received the vaccine	NA
Recommendations from professionals	Recommended by the expert advisory panel of the government	Recommended by the general physicians	NA
Venue for vaccination	Community center	Healthcare facilities	NA
Quarantine for vaccinated travelers	At least 14-day compulsory quarantine	The 14-day compulsory quarantine can be exempted	At least 14-day compulsory quarantine
Which vaccination plan would you like to choose?	Vaccination plan 1: □	Vaccination plan 2: □	Do not accept both: □

Along with the DCE questions, the questionnaire also included (1) experience and behaviors during the COVID-19 pandemic, including perceived susceptibility (four-point scale, “very unlikely,” “unlikely,” “likely,” “very likely”), perceived severity of COVID-19 infection (“If you are infected with COVID- 19, how serious do you think your condition could be?” assessed on a four-point scale, “completely not severe,” “not very severe,” “slightly severe,” “very severe”), and source of COVID-19 vaccine-related information; (2) vaccination-related factors, including previous influenza vaccination acceptance and recommendation from doctors; and (3) basic demographic and socio-economic characteristics, in addition to information about having chronic diseases. Selection of variables follows the constructs of health belief model (38) and the factors that were reported to be associated with vaccine acceptance in previous studies (11, 14–20). The questions for experience and behaviors during the pandemic were designed with reference to a local survey for working-age population (31, 39), which were designed and localized based on WHO guidelines for COVID-19 workplace prevention (40) and validated instruments for knowledge, attitude and practice during Ebola epidemics (41). The vaccination-related questions were designed based on a local vaccine-related survey on healthcare workers (19) and another survey on working population (11). The Cronbach's alpha of the questionnaire was estimated, and found relatively high internal consistency of vaccination-related behaviors and perceptions (α = 0.76) and level of attention to COVID-19 related information (α = 0.72) (42). Prior to the survey, five adults were selected by convenience sampling and invited to conduct a pilot interview to provide feedback on the questionnaire, especially the questions in the DCE section. Four public health professionals were invited to assess and provide suggestions to improve content validity of the questions. The wording was refined based on the feedback, if necessary.

### Statistical Analysis

The demographic, socioeconomic, and health-related characteristics of the respondents are described. To make the study sample comparable to the general working-age population, the sample was standardized for the DCE analysis using a direct method based on the age and sex distribution of the Hong Kong population aged 18–64 years (43). In the analysis, those who chose the opt-out options in all the eight choice sets were defined as “vaccine refusal irrespective of vaccination attributes.” A two-step analytical method was applied in the DCE analysis. First, a multiple logistic regression was used to examine the association between refusal irrespective of attributes and individual-level characteristics including demographic, socio-economic status, chronic conditions, previous vaccination behaviors, and other experiences during the pandemic. The independent variables of the regression were selected based on the hypothesis no. 1 that is specified in the Introduction section, which hypothesized that these factors may be associated with vaccine acceptance based on previous findings. Second, after excluding those who refused vaccination irrespective of attributes, a nested logistic model (NLM) was used to determine the effect of vaccination attributes on acceptance as well as the association between vaccine acceptance and individual-level characteristics, where the dependent variable was the binary choice (0 = “not choose,” 1 = “choose”) for each of the alternatives, and the independent variables were vaccination attributes and individual-level factors. Alternative specific constants that indicated whether the first or the second vaccination alternative was selected were applied to the model to adjust the position bias of the alternatives. The individual-level factors included age, sex, education, occupation, income, chronic condition, perceived risks and severity, previous influenza vaccination, and information sources. The model is specified as follows:


(2) $$\begin{array}{r} U_{iv}=V_{iv}+\;\varepsilon_{i} \end{array}$$

where the utility (U_v_) for acceptance of the COVID-19 vaccine of individual i consists of a deterministic component (V_v_) and a stochastic component (ε). The deterministic component is a function of individual-level factors (X) and the utility (V_a|v_) derived from the attributes of the chosen vaccination programme (A_k_), given that the individual has decided to accept the COVID-19 vaccine:


(3) $$\begin{array}{r} V_{iv}=\delta X_{i}+\lambda \cdot E\left[ln\Sigma e^{V_{ia|v}}\right] \end{array}$$

(4) $$\begin{array}{r} V_{ia|v}=\overset{l}{\underset{k=0}{\sum }}{\beta_{ik}A}_{k} \end{array}$$

δ, β, and λ are the parameters to be estimated. The NLM is based on the random utility theory, where the decision-making process involves two decisions: (1) whether to accept the vaccine (opt-out or non-opt-out), and (2) and which vaccines to accept (alternative A or B). λ is the inclusive-valued parameter that denotes the relationship between the two decisions and lies between 0 and 1. There are two special occasions. If the change in the probability of choosing alternative A only affects B, but not the probability of choosing opt-out (λ = 0), it means that the two decisions are independent of each other and separate logistic models should be applied to modeling the two decisions. However, if the change in the probability of choosing alternative A proportionately affects the probability of choosing B and opt-out (λ = 1), which supports the independence of the irrelevant alternative (IIA) assumption, a multinomial logit model should be applied. The NLM considers a wide spectrum of the levels of influence (0 ≤ λ ≤ 1) of the expected utility of accepting the vaccination alternatives on vaccine acceptance in DCE choice sets, which derive more accurate estimates for the effect of the attributes (44, 45).

Moreover, the NLM model was also performed among subgroups with different sociodemographic and health-related characteristics to identify the difference in the effect of these vaccination attributes on its acceptance among these subgroups. Mixed logit model (MIXL) was also performed as sensitivity analysis (results in Tables A2-3 in Supplementary Material 2). Stata 15.0 was used in statistical analysis.

## Results

### Sample Characteristics

Of the 2,430 invited persons, 2,341 were eligible. Among them, 325 refused the survey and 243 responses were incomplete; thus, 1,773 valid responses were received, with a response rate of 76%. Among the respondents, 61.3% were female, and 43.2% were aged 18–29 years, 34.0% were aged 30–44 years, 18.3% were aged 45–59 years, and 4.5% were aged over 60 years. Over half of the respondents (55.8%) had a bachelor's degree or above, and the monthly income of a similar percentage (55.8%) was HK$30,000. Of these, 10.6% were diagnosed with chronic conditions. Regarding experience during the pandemic, 16.8% knew someone who was diagnosed with COVID-19. Only 7.7% received recommendations for COVID-19 vaccination from doctors (Table 3).

### Response Behaviors

The median survey length was 7 min and 34 s [interquartile range (IQR): 330–658s]. Among the participants, 5% took more than 30 min to answer the questionnaire as the online survey allowed them to log in several times to complete it while recording the duration between their start time and completion time. Only a few participants (4.68%) constantly selected the same vaccination alternative in all eight choice sets (i.e., either all the first alternatives [2.82%] or all the second alternatives [1.86%]) were selected, while 29.0% selected the opt-out option constantly. The percentage of the opt-out selection in the eight choice sets ranged from 43.4 to 46.9%, which was relatively stable. The likelihood of choosing the first vaccination alternative (29.9%) was slightly higher than that of the second alternative (24.8%).

### Vaccine Refusal Irrespective of Vaccination Attributes

The age- and sex-standardized refusal rate, irrespective of vaccination attributes, was 23.6% (Table 3). In the multiple regression (Tables A2-1 in Supplementary Material 2), frequent exposure to COVID-19 vaccine information on social media was associated with refusal [adjusted odds ratio (AOR): 1.60, 95% confidence interval (CI): 1.22–2.10], while exposure to information from family/friends (AOR: 0.71, 95% CI: 0.53–0.95) and government (AOR: 0.66, 95% CI: 0.50–0.87) was associated with a lower likelihood of refusal. Recommendations for vaccination from doctors were also associated with lower chances of refusal (AOR: 0.54, 95% CI: 0.32–0.91).

**Table 3:** Sample characteristics and rate of vaccine refusal by subgroups.

	**Vaccine refusal irrespective of attributes**, ***n*** **(%)**		**Accept at least one vaccine** **alternative in DCE**, ***n*** **(%)**		**Overall, *n* (%)**	***P*-value**
	**Unstandardized rate**	**Standardized rate^**a**^**	**Unstandardized rate**	**Standardized rate^**a**^**		
**Age group (years)**						
18–29	288 (37.7)	36.8	477 (62.4)	63.2	765 (43.2)	<0.001^**^
30–44	164 (27.2)	27.1	439 (72.8)	72.9	603 (34.0)	
45–59	49 (15.1)	14.5	276 (84.9)	85.5	325 (18.3)	
60–64	13 (16.3)	16.2	67 (83.8)	83.8	80 (4.5)	
**Sex**						
Male	178 (26.0)	21.8	508 (74.1)	78.2	686 (38.7)	0.025^*^
Female	336 (30.9)	25.1	751 (69.1)	75.0	1,087 (61.3)	
**Education level**						
Below bachelor degree	215 (27.5)	22.4	568 (72.5)	77.6	783 (44.2)	0.206
Bachelor degree or above	299 (30.2)	24.9	691 (69.8)	75.1	990 (55.8)	
**Occupation**						
Professional/associated professional	220 (29.6)	23.9	523 (70.4)	76.1	743 (41.9)	<0.001^**^
Clerical/service/sale	129 (32.0)	27.2	274 (68.0)	72.8	403 (22.7)	
Blue-collar worker	28 (22.8)	20.8	95 (77.2)	79.2	123 (6.9)	
Unemployed	23 (34.3)	30.0	44 (65.7)	70.0	67 (3.8)	
Students/intern	84 (34.2)	33.8	162 (65.9)	66.2	246 (13.9)	
Others	30 (15.7)	14.7	161 (84.3)	85.4	191 (10.8)	
**Monthly household income**						
Below $30,000	214 (27.3)	22.1	570 (72.7)	77.9	784 (44.2)	0.161
$30,000 or above	300 (30.3)	24.8	689 (69.7)	75.2	989 (55.8)	
**Household size**						
Living alone	38 (34.6)	31.5	72 (65.5)	68.5	110 (6.2)	0.258
2–3 ppl	234 (27.6)	21.0	615 (72.4)	79.1	849 (47.9)	
4+ ppl	242 (29.7)	25.4	572 (70.3)	74.6	814 (45.9)	
**Any chronic condition**						
No	468 (29.5)	24.4	1,118 (70.5)	75.6	1,586 (89.5)	0.162
Yes	46 (24.6)	19.1	141 (75.4)	80.9	187 (10.6)	
**Know anyone diagnosed with COVID-19**						
No	416 (28.2)	22.8	1,060 (71.8)	77.2	1,476 (83.3)	0.095
Yes	98 (33.0)	28.3	199 (67.0)	71.7	297 (16.8)	
**Perceived “likely/very likely” to be infected**						
No	302 (29.8)	23.6	710 (70.2)	76.5	1,012 (57.1)	0.362
Yes	212 (27.9)	23.7	549 (72.1)	76.3	761 (42.9)	
**Perceived “slightly severe/very severe” if get infected COVID-19**						
No	264 (30.7)	25.3	597 (69.3)	74.7	861 (48.6)	0.132
Yes	250 (27.4)	22.0	662 (72.6)	78.0	912 (51.4)	
**Previous influenza vaccination**						
No	445 (31.0)	26.3	992 (69.0)	73.7	1,437 (81.1)	<0.001^**^
Yes	69 (20.5)	14.1	267 (79.5)	85.9	336 (19.0)	
**“Usually” received vaccine information from social media**						
No	227 (27.4)	21.4	603 (72.7)	78.6	830 (46.8)	0.153
Yes	287 (30.4)	25.8	656 (69.6)	74.2	943 (53.2)	
**“Usually” received vaccine information from family/friends**						
No	351 (30.0)	24.8	821 (70.1)	75.2	1,172 (66.1)	0.214
Yes	163 (27.1)	21.5	438 (72.9)	78.5	601 (33.9)	
**“Usually” received vaccine information from workplace**						
No	333 (30.6)	25.2	757 (69.5)	74.8	1,090 (61.5)	0.067
Yes	181 (26.5)	21.1	502 (73.5)	78.9	683 (38.5)	
**“Usually” received vaccine information from government's official source**						
No	378 (32.8)	28.1	773 (67.2)	71.9	1,151 (64.9)	<0.001^**^
Yes	136 (21.9)	17.4	486 (78.1)	82.6	622 (35.1)	
**Doctor's recommendation for vaccination**						
No	489 (29.9)	24.7	1,147 (70.1)	75.3	1,636 (92.3)	0.004^*^
Yes	25 (18.3)	12.5	112 (81.8)	87.6	137 (7.7)	
**Total**	514 (29.0)	23.6	1,259 (71.0)	76.4	1,773 (100.0)	

* *P < 0.05*,

** *P < 0.001*.

a *The standardized rate were calculated using direct method based on age and sex distribution of Hong Kong working-age population*.

### Effect of Vaccination Attributes on Vaccine Acceptance

The effect of vaccination attributes on acceptance was examined among the sample excluding those who refused vaccination irrespective of the attributes, and reported in Tables 4, 5. Most of the vaccination attributes led to a different likelihood of vaccine acceptance. The efficacy of the vaccine had the strongest effect on acceptance. Compared to a vaccine with a 50% efficacy, a 90 and 70% efficacy could lead to 2.35 times (95% CI: 1.91–2.90) and 1.48 times (95% CI: 1.33–1.65) increase in the likelihood of vaccine acceptance, respectively. For vaccine safety, compared with the rate of 1/10,000 serious adverse events, the rate of 1/100,000 increased 19% (95% CI: 1.12–1.26) of the likelihood of vaccine acceptance. Independent of the efficacy and safety of the vaccine, the likelihood of acceptance of BioNTech was 31% more than Sinovac, while AstraZeneca had a reduced likelihood by 11%. Apart from the characteristics, a friend/colleague receiving the vaccine increased the likelihood by 13% (95% CI: 1.06–1.20); however, whether a family member received the vaccine did not make a difference. Recommendations made by general physicians and an expert advisory panel of the government had similar effects on improving vaccine uptake (AOR: 1.00, 95% CI: 0.95–1.04). As for the venue for vaccination, compared with community centers (i.e., existing arrangement), vaccination at housing or workplaces had a similar influence on vaccine acceptance (AOR: 1.05, 95% CI: 0.99–1.11), while vaccination at healthcare facilities could reduce the likelihood of vaccine acceptance by 12% (95% CI: 0.82–0.94). The exemption of 14-day compulsory quarantine for vaccinated travelers increased by 31% (95% CI: 1.21–1.41).

**Table 4:** Nested logit model results for the effect of vaccination attributes on vaccine acceptance elicited in discrete choice experiment.

**Attributes of the vaccination programme**	**Vaccine acceptance (*****n*** **=** **1,259)**^**a**^	
	**AOR^***b***^**	**95%CI^***b***^**
**Brand (“Sinovac” as reference)**		
BioNTech	1.31^**^	(1.21, 1.41)
AstraZeneca	0.89^*^	(0.82, 0.96)
**Efficacy (“50%” as reference)**		
Reduce 70% infections	1.48^**^	(1.33, 1.65)
Reduce 90% infections	2.35^**^	(1.91, 2.90)
**Serious adverse event (“1/10,000 ppl” as reference)**		
1/100,000 ppl	1.19^**^	(1.12, 1.26)
**Vaccine uptake of others (“No known people uptake the vaccine” as reference)**		
Friends/colleagues received	1.13^**^	(1.06, 1.20)
Family members received	1.06	(1.00, 1.13)
**Recommendations from experts (“From general physicians” as reference)**		
From expert advisory panel of the government	1.00	(0.95, 1.04)
**Venue for vaccination (“Community center” as reference)**		
Healthcare facilities	0.88^**^	(0.82, 0.94)
Housing estate/workplace	1.05	(0.99, 1.11)
**Quarantine arrangement for vaccinated traveler (“Compulsory quarantine required” as reference)**		
14-day compulsory quarantine can be exempted	1.31^**^	(1.21, 1.41)
**Alternative specific constant (first vaccination alternative as reference)**		
Second vaccination alternative	0.88	(0.83, 0.93)
**Inclusive-valued parameter** **λ**	0.85	(0.64, 1.07)

* *P < 0.05*,

** *P < 0.001*.

a *The nested logit model was performed among respondents who accept at least one vaccination alternative in discrete choice experiment, n = 1,259*.

b *AOR, adjusted odds ratio; CI, confidence interval*.

**Table 5:** The association between individual-level factors and vaccine acceptance among those accepted at least one of the DCE choice tasks.

**Individual-level factors**	**Vaccine acceptance (*****n*** **=** **1,259)^a^**	
	**AOR^***b***^**	**95%CI^***b***^**
**Age group (18–29 yrs as reference)**		
30–44 yrs	1.43^**^	(1.22, 1.66)
45–59 yrs	2.11^**^	(1.79, 2.49)
60–64 yrs	1.40^*^	(1.11, 1.75)
Female (male as reference)	0.50^**^	(0.56, 0.45)
**Education (Below Bachelor degree as reference)**		
Bachelor degree or above	0.80^**^	(0.71, 0.90)
**Occupation (Professional/associate professional as reference)**		
Clerical/service/sales	1.33^**^	(1.15, 1.54)
Blue-collar worker	1.10	(0.89, 1.35)
Unemployed	2.61^**^	(1.86, 3.65)
Students/intern	1.43^*^	(1.14, 1.79)
Others	2.08^**^	(1.72, 2.51)
Monthly household income > HK$30,000	1.40^**^	(1.24, 1.57)
With any chronic condition	1.14	(0.97, 1.34)
Know anyone diagnosed with COVID-19	1.12	(0.97, 1.29)
Perceived “likely/very likely” to be infected	1.08	(0.97, 1.20)
Perceived “slightly severe/very severe” if get infected COVID-19	1.24^**^	(1.11, 1.37)
Previous influenza vaccination	1.26^**^	(1.11, 1.44)
“Usually” received vaccine information from social media	1.01	(0.89, 1.14)
“Usually” received vaccine information from workplace	1.42^**^	(1.24, 1.61)
“Usually” received vaccine information from family/friends	0.96	(0.85, 1.10)
“Usually” received vaccine information from government's official source	1.68^**^	(1.49, 1.89)
Doctor's recommendation for vaccination	0.97	(0.81, 1.16)

* *P < 0.05*,

** *P < 0.001*.

a *The nested logit model was performed among respondents who accept at least one vaccination alternative in discrete choice experiment, n = 1,259*.

b *AOR, adjusted odds ratio; CI, confidence interval*.

### Subgroup Analysis

The results of subgroup analysis are shown in Supplementary Material 3. The effect of vaccination attributes on vaccine acceptance was different among the respondents of different ages, incomes, chronic conditions, and previous influenza vaccine injections. Those aged over 60 years were more likely to accept Sinovac (AOR for BioNTech: 0.40, 95% CI: 0.27–0.61) independent of the efficacy and safety, and attached a much greater value to efficacy (AOR for 90% efficacy: 5.46, 95% CI: 2.85–10.48) independent of the brand. However, younger people preferred BioNTech over Sinovac and AstraZeneca, and had a relatively lower preference for high efficacy than older persons. Older adults were less likely to accept the vaccine if their family members had (AOR: 0.55, 95% CI: 0.35–0.86); however, the acceptance of family members contributed to a higher acceptance among people aged 30–59 years. People with lower incomes had similar preferences for Sinovac and BioNTech (AOR: 1.04, 95% CI: 0.97–1.11), while those with higher incomes were much more likely to accept BioNTech (AOR: 1.64, 95% CI: 1.42–1.89) and attached a slightly higher value to vaccine efficacy and safety than those with lower incomes. People with chronic conditions were found to focus more on the efficacy of the vaccine (AOR for 90% efficacy: 5.19, 95% CI: 2.57–10.48) than its safety (AOR: 1.18, 95% CI: 0.99–1.40) and other arrangements. Regarding acceptance of the influenza vaccine in the last year, those with this experience tended to focus on the efficacy and safety of the vaccine, and were less likely to be influenced by the vaccine acceptance of other people than others without acceptance of the influenza vaccine.

## Discussion

This study is one of the limited DCE studies highlighting the effect of vaccination attributes on the acceptance of the COVID-19 vaccine other than its association with individual-level factors. The DCE results indicated that vaccine acceptance is substantially and independently affected by the brand, efficacy, and safety of the vaccine, as well as multiple factors related to the implementation of the vaccination programmes, and the effects of the attributes were different among people with different socio-demographic and health-related backgrounds.

From the DCE, it was found that the vaccine refusal rate irrespective of the attributes was 23.6% (during the fourth local epidemic wave), which was similar to the standardized vaccine refusal rates found in Hong Kong in the second half of 2020 (during the third local epidemic wave), which were 21.2% (10) and 21.5% (11). The main reasons for vaccine refusal reported by the participants were lack of confidence in the effectiveness and safety of the vaccine (Supplementary Material 2). Among those participants who refused all 16 vaccination alternatives, only 3.5% would consider accepting the vaccination if the government relaxed the restrictions for physical distancing for COVID-19 containment when the total vaccination coverage reached 70%, an approximate level that was estimated to be a threshold for herd immunity (7). This indicates that collective interests are unlikely to alter the individual vaccination behaviors of individuals who refuse vaccination. This was consistent with the findings reported in the DCE study on COVID-19 vaccination in France, which found no significant association between vaccine refusal and the information that 50% vaccination coverage is needed for herd immunity (22). Female participants were more likely to refuse the vaccine. This is consistent with the findings from a systematic review of vaccination acceptance studies from 33 countries in which males had higher acceptance rates in 15 countries, which was interpreted as the higher perceived risks of COVID-19 infection and lower susceptibility to conspiracy theories about the disease among male (17, 46). Regarding educational level, those with a bachelor degree or above are less likely to refuse the vaccination regardless of the attributes, which is similar to the findings of a global investigation, as they have more knowledge regarding the risk of disease, and side effect and efficacy of the vaccine which may probably reduce their hesitancy (12). However, if they chose to accept at least one of the vaccination alternatives, they were more likely to opt-out in some of the choice tasks. The potential reason for this choice pattern among those with higher educational levels is that their decisions highly depended on the certain attribute levels of the alternatives presented based on their preferences, rather than vague impressions irrespective of the attributes. Along with the association between refusal and individual-level socio-demographic factors, another factor that should be emphasized is that frequent exposure to vaccine-related information on social media rather than from government and in-person communication with family/friends was associated with a greater chance of refusal. Although no causal relationship between the use of social media and vaccine refusal can be determined, this association suggests the importance of accessing reliable information in vaccination promotion. However, it is challenging to ensure that social media provides reliable information and to eliminate the misinformation that could reduce the intention to accept the vaccine (47–50). Therefore, relevant information for vaccination promotion should not only be made available through the government's official channel, but should also be tailored for dissemination through social media and daily communications among people.

Regarding the characteristics of the vaccine, efficacy was found to have the largest independent effect on vaccine acceptance, followed by its brand and safety. Similar findings were reported in previous studies, in which efficacy was the most important attribute for acceptance of the vaccine for COVID-19 or a hypothetical pandemic (23, 51, 52). This finding suggests the importance of disseminating information on vaccine efficacy to the targeted population for their promotion. Participants aged 60 or above and with chronic conditions attached a greater value to efficacy than younger people and those without chronic conditions, which was probably because this group is more likely to have severe COVID-19 infection (53). While efficacy is important in vaccination decision-making for this group, caution needs to be taken in assessing whether an individual is suitable for vaccination based on safety concerns reported in clinical studies (33–36).

The potential exemption of 14-day quarantine for vaccinated travelers was a key attribute in improving vaccine acceptance, which, to our knowledge, has not been examined in previous studies. This finding implies that these kind of relaxation measures, such as travel and social distancing constraints, could be considered by the authorities as incentives to increase vaccine acceptance. The US Center for Disease Control and Prevention has updated its guidance on 2 April for fully vaccinated people to travel within the country without prior tests or return to the US without self-quarantine based on the real-world effectiveness of the vaccination (54). Nonetheless, more studies should be conducted to weigh the potential risk increment of imported COVID-19 cases posed by this strategy and the benefits of quicker and greater vaccine uptake before its implementation. Moreover, vaccination at healthcare facilities could reduce people's acceptance of vaccination compared with community halls, while vaccination at housing estate or workplaces could improve vaccine acceptance among people aged 60 years or above. This is comparable to another DCE study in Australia which found that vaccination at pharmacies instead of at hospitals or doctors' offices could improve vaccine acceptance (27), while it did not test the preference for venues other than healthcare-related locations. The findings indicated that authorities should consider providing vaccination services at community halls or even housing estate or workplaces, rather than healthcare facilities, to make it more convenient for the public to access the vaccine. As for recommendations from healthcare professionals, a study in the US (24) found that recommendations from professional institutions (CDC and WHO) have a greater effect in improving vaccine acceptance than politicians, while our study found recommendations from physicians could have pose similar impact on vaccine acceptance as the healthcare experts representing the government. This highlights the necessity of involving general physicians in vaccination promotions.

This study observed that the peer acceptance influence of friends/colleagues could improve the vaccination uptake of the participants, while the acceptance of family members had little effect. This is similar to a study conducted in mainland China, where vaccine acceptance increased with the percentage of acquaintances who were vaccinated. The difference is that our study suggests that, on average, people are generally more likely to be influenced by peers of a similar age group (i.e., friends) in deciding whether to be vaccinated, which has been reported in previous studies on the social influence of risk perceptions among adolescents (55). It was also found that the influence of vaccination uptake by friends or colleagues was not consistent between people aged 60 years or above and those aged below 60 years. A lower likelihood of vaccine acceptance was found among people aged above 60 years when their family members accepted the vaccine, while the acceptance of family members, as well as friends/colleagues, contributed to a greater chance of acceptance among those aged <60 years. This finding supports the free-rider hypothesis for vaccination among older adults, which was reported in studies on influenza vaccine acceptance among the general population and healthcare workers. It states that people will avoid vaccination when its perceived coverage is high enough to achieve herd immunity in their social network (56, 57). It also emphasizes the influence of social norms on the vaccine uptake among people aged below 60 years, which means the vaccination behaviors of peers may improve the uptake; this has been reported in previous studies on vaccination (58–60). This disparity might be because older adults aged over 60 years are less sensitive to peer influence. They might also perceive a greater risk of vaccination side effects due to the chronic conditions that they have, which makes them reluctant to be vaccinated when they perceive lower COVID-19 infection risks as the people around them are vaccinated. However, the disparity across age groups requires further investigation and evidence.

At the time of the survey, the fourth wave of COVID-19 in Hong Kong was under control, the daily new cases were relatively low, ranging from 22 to 32 cases per day, and the vaccination programme had commenced for 1 week (13). Despite the low level of daily confirmed cases, vaccination is crucial for the sustainable control of COVID-19. By 8 April 2020 ~7.9% of the Hong Kong population had received at least one dose of the vaccine, and ~2.2% received two doses (13), which was far from the 70% coverage rate needed to achieve herd immunity (7). This DCE study examined the causal relationship between the vaccination attributes identified based on TDF and acceptance of the vaccination. The study results highlight the importance of efficacy and brand in decision-making, which supports findings from other DCE studies that vaccine effectiveness and efficacy, as well as place of origin have higher priorities than other characteristics in deciding the vaccines (21, 22, 25). It also suggested that the involvement of general physicians in providing recommendations for vaccination, recommendations from experts in government advisory panels, the provision of vaccination at housing estate or workplaces, and exemption of quarantine for vaccinated travelers can improve the vaccination of the general public, which provided evidence for how the change of these modifiable factors of vaccination could improve the willingness and the potential effectiveness of such measures, as suggested by the previous studies. The policy intervention on the vaccination and other public health measures also changes over time with the progress of pandemic. Most of existing studies were conducted when the vaccines had not been available to the public yet. Thus, including the attributes derived from an update qualitative study for the DCE conducted under more realistic scenarios when vaccination programme has commenced would increase the capacity of DCE to understand people's preference and decision-making process. Dissemination of official information through social media can also be considered in providing reliable information and reducing vaccine refusal. For people aged 60 years or above, with chronic conditions and having previous experience of influenza vaccination, efficacy and safety of the vaccine are more important, so relevant information should be made available to them, particularly in detail, and a pre-vaccination screening to examine whether their health conditions are suitable for vaccination could be helpful. These findings can be used to inform the formulation of COVID-19 vaccination promotion strategies for either designing or implementing public health policies and interventions to improve vaccination rollout speed.

A few limitations of this study need to be noted. First, the findings from DCE could vary during the progress of the vaccination programme, and could be affected by changes in local epidemic situations and newly reported vaccine-related incidents, or serious adverse events locally and globally. Nonetheless, this study forms a baseline of vaccination preference for the working-age population in Hong Kong at the beginning of COVID-19 vaccination, and further follow-up surveys collecting the trend of vaccine acceptance or uptake of the participants from the current study or other studies at different time points should be considered to assess the influence of relevant incidents or events on their acceptance. Secondly, the reason for using a vaccine brand as an attribute is that it has been considered as an important factor in decision-making regarding vaccination in the prior qualitative interview and public discussion, and the effect of vaccine efficacy and safety on acceptance should be tested independently by controlling its brand, which captures people's trust in the vaccines. However, preference for a vaccine brand can be interpreted in different ways, including a preference for their place of origin, manufacturers, the type of vaccine technologies, and/or a combination of incidents related to that brand. Although preference for these factors cannot be differentiated from this study, further studies can be conducted on this matter. Thirdly, there are some limitations in the design of the DCE. First, exercise tasks for the participants and confirmation of the choices of the exercise tasks should be provided in DCE tasks to improve participants' understanding of the formal tasks. Second, the order of the choice tasks, alternatives, and attributes should be randomized in the survey for different participants to reduce the influence of the positions of the DCE elements on responses, while this function cannot be achieved in the online platform for the survey. Finally, the reliability and validity of this questionnaire was not systematically examined prior to the survey due to limitation in the study design, including test-retest reliability, inter-rater reliability and criterion validity. Nevertheless, since the questionnaire was designed based on validated tools in previous studies and have been adopted in local context in a few studies as mentioned in Method section, it can be considered to be reliable and valid in capture information on vaccine acceptance and related factors.

## Conclusion

This study showed that various characteristics of the COVID-19 vaccine and vaccination delivery could substantially influence the acceptance of working-age people, hence affecting the coverage and speed of vaccination rollout. The study findings provide evidence for the use of several strategies to promote vaccination, including provision of vaccination at places more convenient to the public (such as community hall, housing estate, or workplace), involvement of general physicians in vaccine promotion and information dissemination, and exemptions of constraint measures used for COVID-19 prevention for vaccinated people. The free-rider effect was found to be significant among people aged over 60 years, while social norm posed a larger influence among younger people, so dissemination of percentage of vaccinated people as a way to promote vaccination could be more useful among younger population. Frequent exposure to social media might increase vaccine refusal, which is a significant information channel that cannot be neglected in the dissemination of official information regarding vaccination and related incidents. Further studies of respondents can be conducted to gather data about their experience during the vaccination and the potential change in their preference for vaccination.

## Data Availability Statement

Data used in this study cannot be made publicly available for ethical reasons. Public availability of data would compromise confidentiality and privacy of participants.

## Ethics Statement

The studies involving human participants were reviewed and approved by Survey and Behavioral Research Ethics Committee of the Chinese University of Hong Kong. The patients/participants provided their written informed consent to participate in this study.

## Author Contributions

KW conceptualized, designed, implemented the study, performed the data analysis, and drafted the manuscript. ELYW conceptualized, designed, and made critical revisions to the manuscript. AWLC and PSYY contributed to acquisition of the data and data analysis. EKY contributed to conceptualization of the study and made critical revisions to the manuscript. All authors edited and approved the final version of the manuscript.

## Funding

This study was supported by K.S. & Feili Lo Foundation Limited.

## Conflict of Interest

The authors declare that the research was conducted in the absence of any commercial or financial relationships that could be construed as a potential conflict of interest.

## Publisher's Note

All claims expressed in this article are solely those of the authors and do not necessarily represent those of their affiliated organizations, or those of the publisher, the editors and the reviewers. Any product that may be evaluated in this article, or claim that may be made by its manufacturer, is not guaranteed or endorsed by the publisher.
