# Supplementary material for: Influence of Vaccination Characteristics on COVID-19 Vaccine Acceptance Among Working-Age People in Hong Kong, China: A Discrete Choice Experiment
Source: Front Public Health. 2021 Dec 10;9:793533. doi: 10.3389/fpubh.2021.793533 (PMC8702724; doi:10.3389/fpubh.2021.793533)
Supplement: Supplementary file 1 [file Data_Sheet_1.docx]

Supplementary Material 1

# Qualitative interview findings and descriptive framework

1. Results of qualitative interview

Qualitative interviews were conducted among 45 individuals in Hong Kong to inform the design of discrete choice experiment (DCE) during February 2021. Purposive sampling of general population was performed to selected participants. The sample were stratified according to age, whether to have chronic conditions, and willingness to be vaccinated, and quotas were assigned to different strata. The semi-structured interview was guided by the Theoretical Domains Framework (TDF), which comprises of 14 domains that synthesize a number of theories in identifying facilitators and barriers in behavioral change. The facilitators and barriers of vaccination that was discussed by the participants and their frequencies are reported in the table below (Table A1-1).

Table A1-1. Frequently reported facilitators and barriers for COVID-19 vaccination

|  | Facilitators | Barriers |
| --- | --- | --- |
| High frequency  Low frequency | Expected resumption to normal life after vaccination (n=23) | Worry about severe side effects (n=29) |
|  | Influence of healthcare professionals’ suggestion (n=21) | Low confidence in benefits of receiving the vaccine (n=27) |
|  | High confidence in benefits of receiving the vaccine (n=14) | Insufficient data related to the COVID-19 vaccines (n=12) |
|  | Short travel distance of location for vaccination (n=11) | Perceived low protection against COVID-19 (n=10) |
|  | Origins/brands and data transparency (n=9) |  |
|  | Influence of family members, friends, or relatives' suggestions/ their sharing of experience on receiving COVID-19 vaccination (n=9) |  |

1. Descriptive framework

The descriptive framework was determined based on the qualitative interview results and the information provided by the government on its official website of COVID-19 vaccination programme [1]. It can be found from the qualitative interview results that the expected resumption to normal life, suggestions from healthcare professionals, confidence in benefits, worries on severe side effect, convenience of the vaccination location, brand of vaccine, and influence of people around were more frequently reported by the participants in deciding whether or not to be vaccinated. A survey for local working population found a large proportion of people concerning about the effectiveness and safety of the vaccine, and a small proportion of people stating “no time to uptake” [2]. Therefore, the effectiveness and risks of adverse event of vaccination, and the convenience of vaccination along with other factors identified in the qualitative interviews were considered as attributes to construct the choice tasks.

At the time of the DCE study was conducted (February 2021), the fourth wave of local epidemics was coming to an end, and social distance restrictions was reduced gradually [3]. The HKSAR government has announced the vaccination is free of charge, while no incentives in monetary or in other forms were provided to the public at that time [4]. In February 2021, three brands of vaccines were planned to be launched in Hong Kong, and it was unlikely to introduce new vaccines within the year of 2021 [5]. According to the criteria for vaccination of these 3 vaccines, people aged 18 years and above are eligible for vaccination [6-9]. Two doses with around 21-28 days interval need to be administered for any of the three vaccines [6-9]. There were little scheduling difficulties of vaccination in Hong Kong because of the sufficient supply of vaccine and launch of multiple vaccination centres all over the city. These information contributed to the hypothetical scenarios where the participants made the choices in the DCE study.

The levels of the attributes were formed based on the previous research articles, qualitative interview findings, and practice of vaccination programme in Hong Kong, and described in text in the questionnaire. The efficacy and the probability of serious adverse event of the three vaccines has been reported in the articles of phase2/3 clinical trials, which were used to determine the levels of attributes (efficacy and serious adverse event) [6-9]. The venue for vaccination has been announced to be the community halls and private doctors [4]. The “residence estate/workplace” has been added as another attribute level as we would like to test the preference for a more convenient venue. The recommendations for vaccination are usually made by the government expert panels and by the general physicians who have more frequent contact with the publics. The vaccine acceptance can be influenced by vaccine uptake of people around including family members and friends, according to the qualitative interview, and the impact of these two groups of people were examined separately in comparison with “no vaccination of these people”. The exemption of quarantine for cross-border travel to mainland China and to other countries after vaccination was frequently discussed as part of expected resumption to normal life, while the other social distancing restriction measures has already been eased as the daily number of new cases decreased after the fourth wave of local epidemics.

Reference:

[1]. Hong Kong SAR Government. COVID-19 vaccination programme Hong Kong2021 [Available from: <https://www.covidvaccine.gov.hk/en/>.

[2]. Wang, K,Wong, EL,Ho, KF,Cheung, AW,Yau, PS,Dong, D, et al. Change of Willingness to Accept COVID-19 Vaccine and Reasons of Vaccine Hesitancy of Working People at Different Waves of Local Epidemic in Hong Kong, China: Repeated Cross-Sectional Surveys. Vaccines (Basel). 2021;9(1).

[3]. Choy, G,Ting, V. Hong Kong to ease social-distancing rules for first time in three months since Covid-19 fourth wave hit; city logs eight new cases Hong Kong: South China Morning Post; 2021 [Available from: <https://www.scmp.com/news/hong-kong/health-environment/article/3121863/hong-kong-facing-fewer-10-covid-19-cases>.

[4]. Hong Kong SAR Government. COVID-19 vaccination programme - About the programme Hong Kong2021 [Available from: <https://www.covidvaccine.gov.hk/en/programme>.

[5]. Choy, G,Ting, V. What do we know about the different Covid-19 vaccines for Hong Kong, and which one should you take? Hong Kong: South China Morning Post; 2021 [Available from: <https://www.scmp.com/news/hong-kong/health-environment/article/3122085/what-do-we-know-about-different-covid-19-vaccines>.

[6]. Voysey, M,Clemens, SAC,Madhi, SA,Weckx, LY,Folegatti, PM,Aley, PK, et al. Safety and efficacy of the ChAdOx1 nCoV-19 vaccine (AZD1222) against SARS-CoV-2: an interim analysis of four randomised controlled trials in Brazil, South Africa, and the UK. The Lancet. 2021;397(10269):99-111.

[7]. Polack, FP,Thomas, SJ,Kitchin, N,Absalon, J,Gurtman, A,Lockhart, S, et al. Safety and efficacy of the BNT162b2 mRNA Covid-19 vaccine. New England Journal of Medicine. 2020.

[8]. Zhang, Y,Zeng, G,Pan, H,Li, C,Hu, Y,Chu, K, et al. Safety, tolerability, and immunogenicity of an inactivated SARS-CoV-2 vaccine in healthy adults aged 18–59 years: a randomised, double-blind, placebo-controlled, phase 1/2 clinical trial. The Lancet infectious diseases. 2021;21(2):181-92.

[9]. Wu, Z,Hu, Y,Xu, M,Chen, Z,Yang, W,Jiang, Z, et al. Safety, tolerability, and immunogenicity of an inactivated SARS-CoV-2 vaccine (CoronaVac) in healthy adults aged 60 years and older: a randomised, double-blind, placebo-controlled, phase 1/2 clinical trial. The Lancet Infectious Diseases. 2021;21(6):803-12.
