# Supplementary material for: Influence of Vaccination Characteristics on COVID-19 Vaccine Acceptance Among Working-Age People in Hong Kong, China: A Discrete Choice Experiment
Source: Front Public Health. 2021 Dec 10;9:793533. doi: 10.3389/fpubh.2021.793533 (PMC8702724; doi:10.3389/fpubh.2021.793533)
Supplement: Supplementary file 2 [file Data_Sheet_2.docx]

Supplementary Material 2

**Vaccine refusal irrespective of vaccination attributes and sensitivity analysis**

1. **Supplementary results on vaccine refusal irrespective of vaccination attributes**

A multiple logistic regression was performed to examine the association between refusal irrespective of attributes and individual-level characteristics including demographic, socio-economic status, chronic conditions, previous vaccination behaviours, and other experience during the pandemic. The participants who chose the opt-out options in all of the eight choice sets in their questionnaire were defined as “vaccine refusal irrespective of vaccination attributes”.

In the multiple logistic regression (Table A2-1), vaccination refusal was found associated with younger age (30-64 years: adjusted odds ratio [AOR] range: 0.31-0.64), female (AOR: 1.34, 95% confidence interval [CI]: 1.05-1.71), below bachelor degree (bachelor degree or above: AOR: 0.75, 95% confidence interval [CI]: 0.56-0.99), and higher income (AOR: 1.33, 95% confidence interval [CI]: 1.02-1.73). Those who received influenza vaccination in the 2020 were less likely to refuse COVID-19 vaccination (AOR: 0.55, 95% confidence interval [CI]: 0.40-0.76). Those who usually received vaccine information from family/friends (AOR: 0.71, 95% confidence interval [CI]: 0.53-0.95) and/or government’s official source (AOR: 0.66, 95% confidence interval [CI]: 0.50-0.87), and those received recommendations for vaccination from doctors (AOR: 0.54, 95% confidence interval [CI]: 0.33-0.91) were less likely to refuse vaccination, while those who usually received information from social media (AOR: 1.60, 95% confidence interval [CI]: 1.22-2.10) were more likely to refuse it.

The participants also reported their reasons for vaccine refusal in the survey (Table A2-2). It was found that most of them refused vaccination as they did not have confidence in safety (79.77%) and effectiveness (77.82%) of the vaccine. A number of participants did not trusting the manufacturers (46.30%) and/or believe vaccination is not necessary as keeping personal hygiene is enough (36.96%). A very small proportion of participants reported that there is no time for vaccination (2.92%), or the place for vaccination is inconvenient (3.50%). The findings indicated that the lack of confidence to the vaccines were most important reasons for vaccine refusal.

Table A2-1. Logistic regression results for association between vaccine refusal irrespective of vaccination attributes and social-demographical factors and experience during the pandemic

|  |  | Vaccine refusal irrespective of attributes | |
| --- | --- | --- | --- |
|  |  | AOR^1^ | 95%CI^1^ |
| **Age group (18-29 yrs as reference)** | | |  |
| 30-44 yrs |  | 0.64* | (0.46, 0.88) |
| 45-59 yrs |  | 0.31** | (0.22, 0.46) |
| 60-64 yrs |  | 0.52* | (0.30, 0.88) |
| **Female (male as reference)** | | 1.34* | (1.05, 1.71) |
| **Education (Below Bachelor degree as reference)** | | |  |
| Bachelor degree or above | | 0.75* | (0.56, 0.99) |
| **Occupation (Professional/associate professional as reference)** | | | |
| Clerical/service/sales | | 1.04 | (0.75, 1.43) |
| Blue-collar worker | | 1.06 | (0.66, 1.70) |
| Unemployed |  | 1.29 | (0.70, 2.37) |
| Students/intern | | 0.91 | (0.56, 1.47) |
| Others |  | 0.70 | (0.45, 1.10) |
| **Monthly household income > HK$30,000** | | 1.33* | (1.02, 1.73) |
| **With any chronic condition** | | 1.12 | (0.78, 1.60) |
| **Know anyone diagnosed with COVID-19** | | 1.14 | (0.84, 1.56) |
| **Perceived "likely/very likely" to be infected** | | 1.10 | (0.86, 1.39) |
| **Perceived "slightly severe/very severe" if get infected COVID-19** | | 0.86 | (0.68, 1.10) |
| **Previous influenza vaccination** | | 0.55** | (0.40, 0.76) |
| **"Usually" received vaccine information from social media** | | 1.60* | (1.22, 2.10) |
| **"Usually" received vaccine information from workplace** | | 1.04 | (0.78, 1.39) |
| **"Usually" received vaccine information from family/friends** | | 0.71* | (0.53, 0.95) |
| **"Usually" received vaccine information from government's official source** | | 0.66* | (0.50, 0.87) |
| **Doctor's recommendation for vaccination** | | 0.54* | (0.33, 0.91) |

Note: *P<0.05, ** P<0.001.

1. AOR: adjusted odds ratio; CI: confidence interval

Table A2-2. Reasons for vaccine refusal irrespective of vaccination attributes

| **Reasons for refusal** | **N** | **%** |
| --- | --- | --- |
| No confidence in safety of the vaccines | 410 | 79.77 |
| No confidence in effectiveness of the vaccines | 400 | 77.82 |
| Do not trust the manufacturers | 238 | 46.30 |
| Paying attention to personal hygiene is enough, no need to be vaccinated | 190 | 36.96 |
| Inconvenience place of vaccination | 18 | 3.50 |
| No time for vaccination | 15 | 2.92 |
| Total | 514 | 100 |

1. Sensitivity analysis on preference for vaccination attributes using mixed logit model (MIXL)

A MIXL model was applied to analyzing the preference of participants for vaccination attributes as sensitivity analysis. MIXL model takes into account preference heterogeneity across individual in estimating the preference weightings. Table A2-3 shows the results. It revealed that Efficacy is the most important attribute in choosing the vaccine. The participants preferred for BioNTech over Sinovac and AstraZeneca independently from their efficacy and safety characteristics. Knowing family members or friends/colleagues receiving the vaccine, and exemption of 14-day quarantine for travelers could improve the likelihood of vaccination, while vaccination at healthcare facilities may reduce the likelihood of vaccination. Recommendations for vaccination from general physicians or from expert advisory panel of the government did not make a difference. The results estimated from MIXL is similar to the results estimated from nested logit model (NLM) as the primary analytical method.

Table A2-3. MIXL model results for preference for vaccination attributes

| Attributes of the vaccination programme | Coefficient (Mean) | 95%CI |  | SD | 95%CI |
| --- | --- | --- | --- | --- | --- |
| **Brand ("Sinovac" as reference)** | |  |  |  |  |
| BioNTech | 0.21** | (0.12, 0.30) |  | 1.33** | (1.23, 1.44) |
| AstraZeneca | -0.23** | (-0.33, -0.12) |  | 1.09** | (0.94, 1.24) |
| **Efficacy ("50%" as reference)** | |  |  |  |  |
| Reduce 70% infections | 0.61** | (0.53, 0.68) |  | 0.21* | (-0.43, 0.00) |
| Reduce 90% infections | 1.41** | (1.31, 1.51) |  | 0.77** | (0.65, 0.89) |
| **Serious adverse event ("1/10,000 ppl" as reference)** | | | |  |  |
| 1/100,000 ppl | 0.21** | (0.15, 0.27) |  | 0.48** | (0.34, 0.62) |
| **Vaccine uptake of others ("No known people uptake the vaccine" as reference)** | | | | | |
| Friends/colleagues received | 0.25** | (0.17, 0.32) |  | 0.02 | (-0.17, 0.13) |
| Family members received | 0.24** | (0.15, 0.33) |  | 0.04 | (-0.07, 0.16) |
| **Recommendations from experts ("From general physicians" as reference)** | | | | | |
| From expert advisory panel of the government | 0.02 | (-0.04, 0.09) |  | 0.10 | (-0.04, 0.24) |
| **Venue for vaccination ("Community centre" as reference)** | | | | |  |
| Healthcare facilities | -0.21** | (-0.30, -0.13) |  | 0.20* | (-0.36, -0.04) |
| Housing estate/workplace | 0.05 | (-0.03, 0.13) |  | 0.14 | (-0.01, 0.29) |
| **Quarantine arrangement for vaccinated traveler ("Compulsory quarantine required" as reference)** | | | | | |
| 14-day compulsory quarantine can be exempted | 0.42** | (0.35, 0.49) |  | 0.48** | (0.35, 0.62) |
| **Opt-out** | 1.21** | (0.77, 1.65) |  | 2.86** | (2.45, 3.27) |
