# Supplementary material for: Influence of Vaccination Characteristics on COVID-19 Vaccine Acceptance Among Working-Age People in Hong Kong, China: A Discrete Choice Experiment
Source: Front Public Health. 2021 Dec 10;9:793533. doi: 10.3389/fpubh.2021.793533 (PMC8702724; doi:10.3389/fpubh.2021.793533)
Supplement: Supplementary file 3 [file Data_Sheet_3.docx]

Supplementary Material 3

**Supplementary figures on subgroup analysis**

Figure A1. Odds ratios of vaccination attributes on vaccine acceptance according to age groups


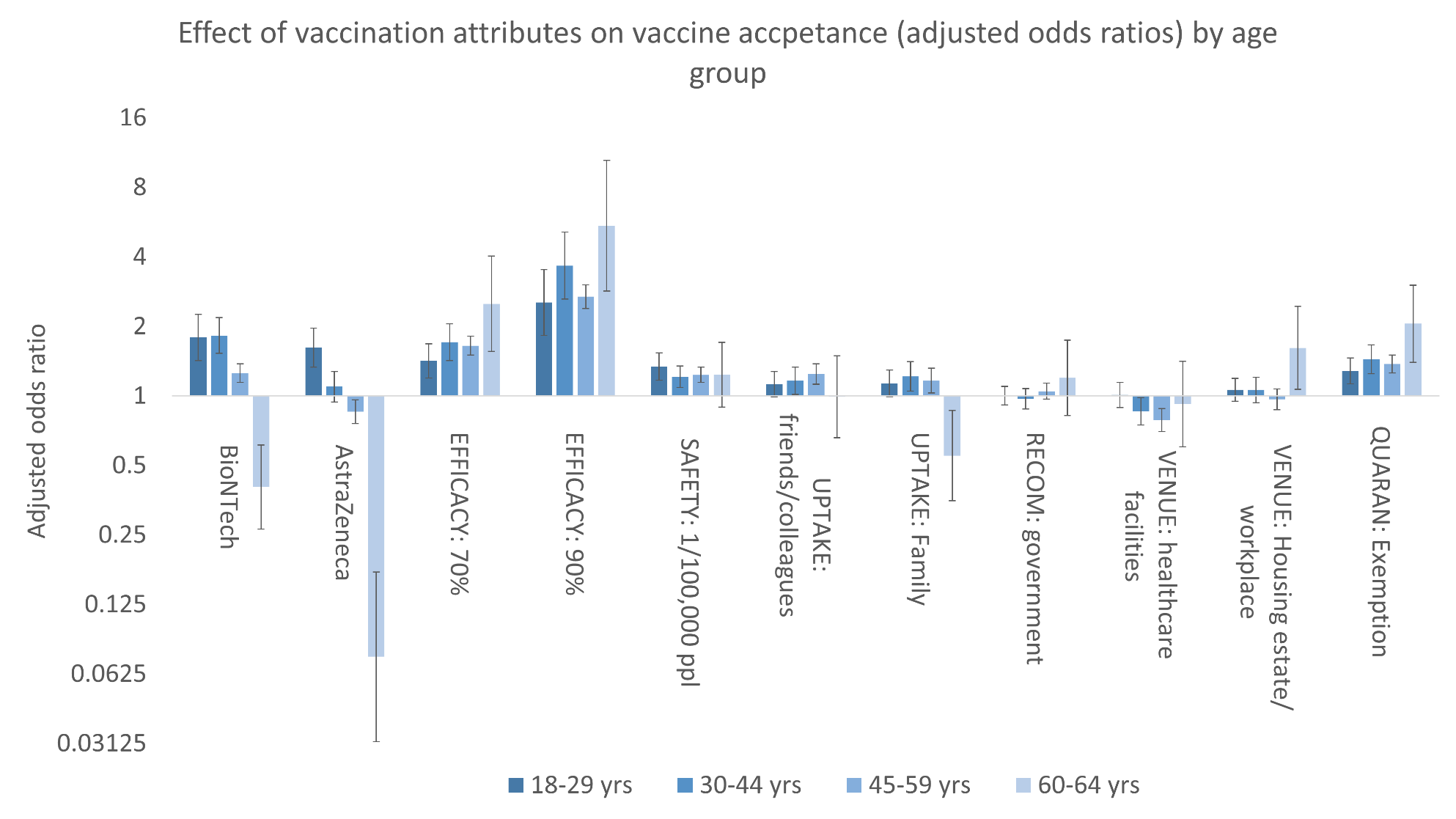


Note: 1. BRAND: vaccine brand (Sinovac as reference), 2. EFFICACY: vaccine efficacy (50% as reference), 3. SAFETY: serious adverse event (1/10,000 as reference), 4. UPTAKE: vaccine uptake of people around (no one around uptake the vaccine as reference), 5. RECOM: recommendation from professionals (recommended by general physician as reference), 6. VENUE: venue for vaccination (community hall as reference), and 7. QUARAN: quarantine exemption for vaccination travelers (no exemption as reference). The error bars presented in the figure are 95% confidence interval of the adjusted odds ratios.

Figure A2. Odds ratios of vaccination attributes on vaccine acceptance according to income level


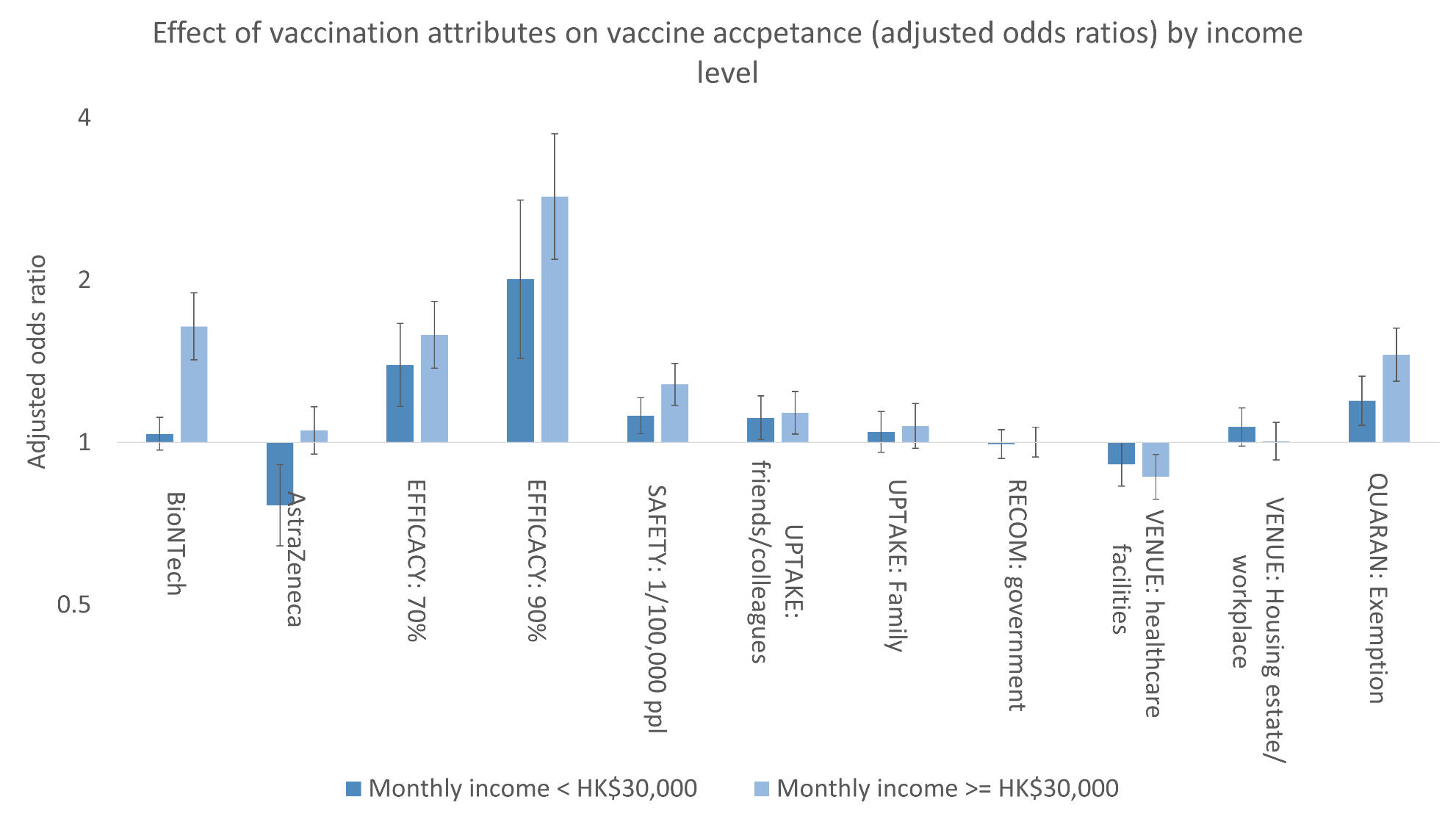


Note: 1. BRAND: vaccine brand (Sinovac as reference), 2. EFFICACY: vaccine efficacy (50% as reference), 3. SAFETY: serious adverse event (1/10,000 as reference), 4. UPTAKE: vaccine uptake of people around (no one around uptake the vaccine as reference), 5. RECOM: recommendation from professionals (recommended by general physician as reference), 6. VENUE: venue for vaccination (community hall as reference), and 7. QUARAN: quarantine exemption for vaccination travelers (no exemption as reference). The error bars presented in the figure are 95% confidence interval of the adjusted odds ratios.

Figure A3. Odds ratios of vaccination attributes on vaccine acceptance according to chronic condition


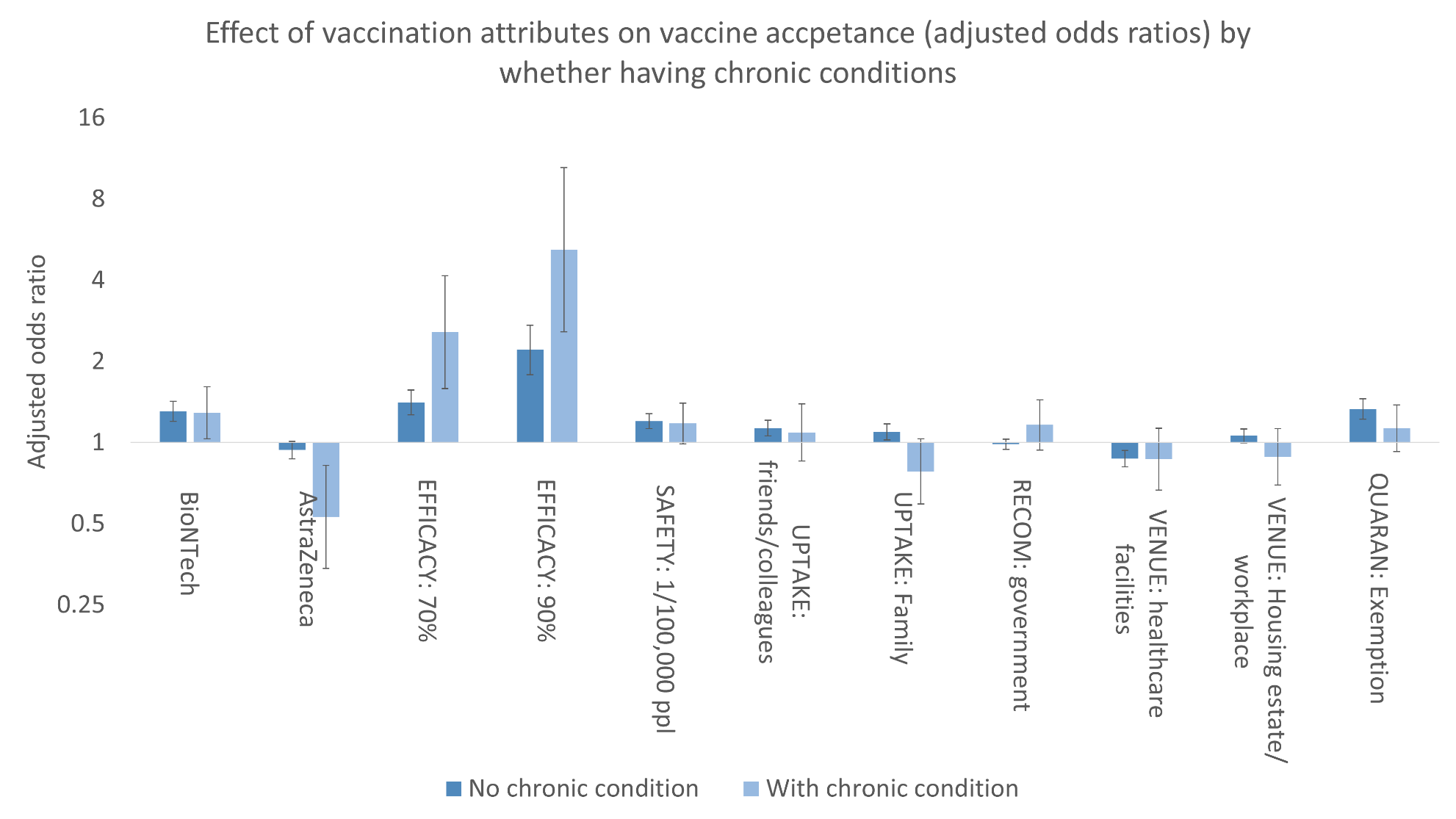


Note: 1. BRAND: vaccine brand (Sinovac as reference), 2. EFFICACY: vaccine efficacy (50% as reference), 3. SAFETY: serious adverse event (1/10,000 as reference), 4. UPTAKE: vaccine uptake of people around (no one around uptake the vaccine as reference), 5. RECOM: recommendation from professionals (recommended by general physician as reference), 6. VENUE: venue for vaccination (community hall as reference), and 7. QUARAN: quarantine exemption for vaccination travelers (no exemption as reference). The error bars presented in the figure are 95% confidence interval of the adjusted odds ratios.

Figure A4. Odds ratios of vaccination attributes on vaccine acceptance according to influenza vaccination


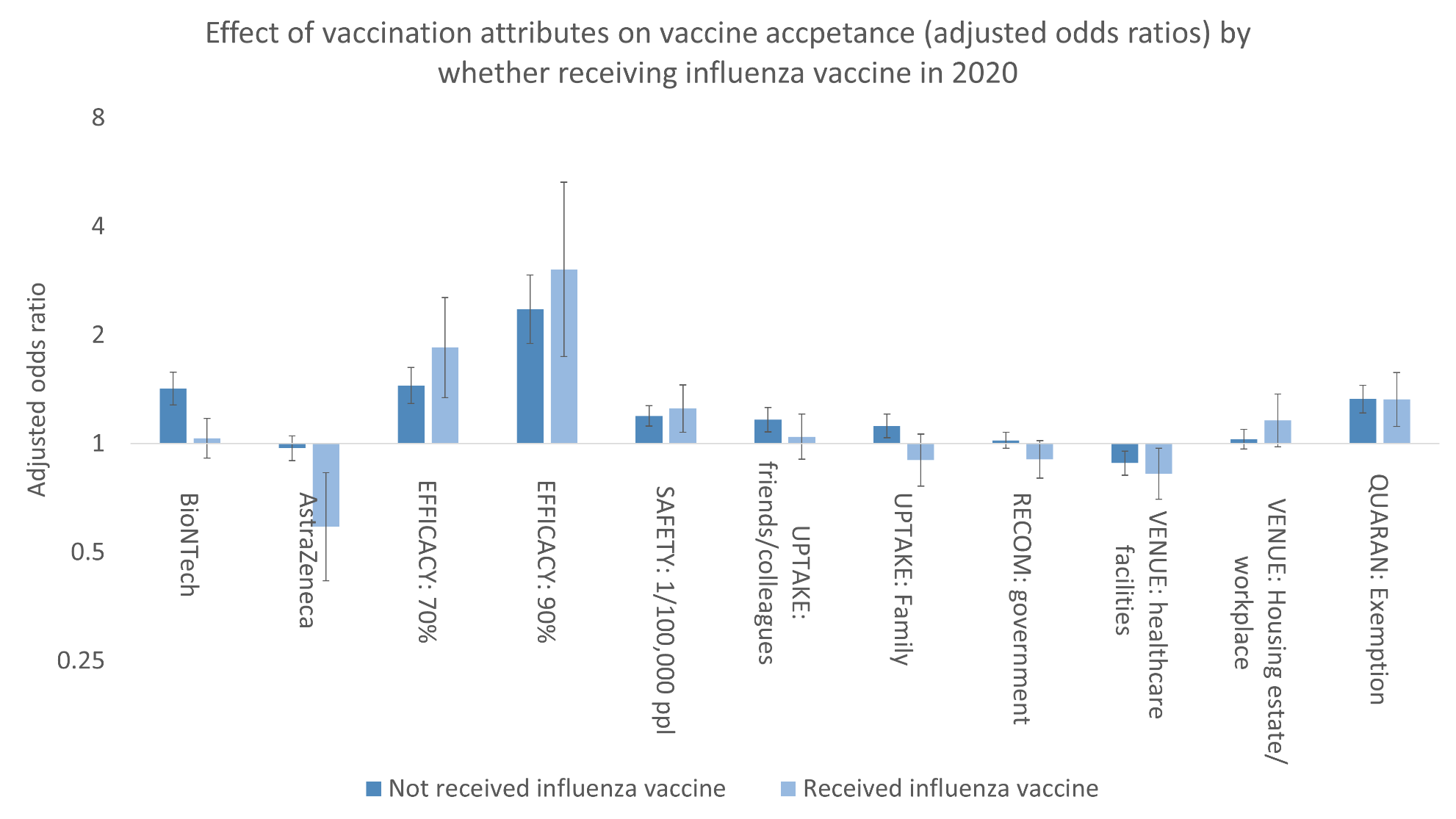


Note: 1. BRAND: vaccine brand (Sinovac as reference), 2. EFFICACY: vaccine efficacy (50% as reference), 3. SAFETY: serious adverse event (1/10,000 as reference), 4. UPTAKE: vaccine uptake of people around (no one around uptake the vaccine as reference), 5. RECOM: recommendation from professionals (recommended by general physician as reference), 6. VENUE: venue for vaccination (community hall as reference), and 7. QUARAN: quarantine exemption for vaccination travelers (no exemption as reference). The error bars presented in the figure are 95% confidence interval of the adjusted odds ratios.
